# Extraction of Teeth

**Published:** 1847-10

**Authors:** J. Lee

**Affiliations:** Camden, S. C.


### ARTICLE III.

*Extraction of Teeth.* By J. LEE, M. D., Camden, S. C.

THE successful extraction of teeth depends much on the manual dexterity of the operator, yet without anatomical knowledge he will often fail, or be compelled to resort to brute force.

The best instrument in the hands of an operator is the one in the use of which he is fully instructed—it is, therefore, of great importance that he should learn with good instruments. I have been in the habit of extracting teeth for twenty-five years, and have used every variety of instrument.

In the office in which I studied medicine, we had a variety of keys and forceps, illy adapted to taking hold, or holding when put on. It would be useless to describe the keys, as they were badly constructed—with revolving fulcrums—spring-triggers—snaps, &c. &c. I found among the discarded instruments the shank of a key slightly bent at the fulcrum—the fulcrum being round, half inch long, three-eighths thick; to this I fitted a handle and a set of claws—it soon became the favorite of the office in preference to others of greater pretensions. While in the practice of medicine, (which includes tooth-pulling in the country,) I used a similar key made for me by Schively of Philadelphia, and which key I yet have; to it I added an India-rubber pad, on a movable button, which I think an improvement.

I used the key many years to extract the molar teeth of both jaws, and if the claw is adapted to the size of the tooth, reasonable success may be expected with it; if the claw is too large the fulcrum will slip down, and the traction will be against the rise of the tooth, it being drawn laterally, so that the alveolus must give way or the tooth be broken off. If the claw is too small the effect will be to snap off the tooth at its neck. But if the claw is of the right size so as to have the edge of the claw horizontally opposite the fulcrum when tightened in the tooth, the fulcrum being adjusted to the palatine side of the upper molar, it will readily follow the revolution of the instrument. In the extraction of a lower molar with the key, the fulcrum should be placed on the labial side of the jaw, and as I am here running against the received notions on this subject, I give the following reasons: The anatomical construction of the lower jaw favors the slipping down of the fulcrum on the inside—the outside widens so as to support it; the inside of the jaw is weak, the outside strong—the inside is liable to splinter, the outside never splinters. From the outside, I think the force required to extract is one-third less, and this I have proved to my satisfaction by drawing similar teeth from the same jaw at the same sitting. The inclination of the teeth inwards, and the outer edge of the jaw being a little highest, favors the rotation outward.

Some ten years since, I came into possession of a set of Cartwright's forceps, or Snell's as they are improperly called, for Snell himself calls them Cartwright's—with these I succeeded so well that I discontinued the use of the key on the upper jaw, but still preferred it in extracting the lower molars. In 1846, I received a pair of molar forceps from Philadelphia, which were an improvement on Cartwright's, claimed by Dr. Harris—they were sent to me by Mr. Charles Abbey. Their great size caused me to lay them by, but meeting with some cases to which I thought they were fitted, I tried them, and since that time I have regarded them as the most valuable extracting instruments in my case, they having superseded the key in the extraction of the lower molars.

Previous to the extraction of a tooth, the gum should be separated from it by a narrow slightly curved lancet—whether you cut up or down is a matter of little consequence, so you completely cut round and down to the bone.

To extract an upper molar with a key, place the patient on a low seat, and stand or set behind him—for the lower, stand before or by the side of the patient—turn the upper teeth inwards, the lower outwards for the molars, the bicuspids of both jaws to be turned inwards. The sapientiæ, generally, from their position, and for want of room require to be turned inwards—but where practicable, they are easiest removed outwardly. The cuspidati and incisores are removed by forceps—those of the lower jaw require to be pressed outward and inward before direct traction is applied—the uppers require a little rotation before traction. Any good pair of forceps, the beaks of which are fitted to the slopes of the teeth may be used. My prejudices are in favor of those manufactured by Mr. Arnold of Baltimore.

All roots, or decaying teeth, causing inflammation of the gums should be removed. Some teeth, apparently past use, will, after killing the nerve, and being filled with tin, cease to be offensive, and last several years. In such teeth I prefer tin to gold, and if asked why, I answer, that ten years experience satisfies me that it is better.

But we find in some mouths the roots of teeth even with the gums, clean and healthy, useful as a chewing surface. Such roots I do not extract—they are useful in keeping the countenance full. They should be extracted previous to setting artificial teeth.

In extracting with the key or forceps, with ordinary care, it is scarcely possible to extract several teeth at once. By permitting the claw to grasp two teeth, or where two are standing together, those before and behind having been previously removed, the alveolus may give way and two come out together.

Miss P. requested a physician to extract the second molar, the first having been removed sometime previous—the alveolus gave way, and the sapientiæ came away with it, a considerable dimple in the cheek was the consequence.

Sometimes the roots of the teeth are united by exostosis, but this is rare—I have seen one case—here two teeth came away when pulling one. It was a case of Dr. T. J. Flinn's, of Darlington, S. C., and is similar to a case published by Dr. Harris. Exostosis, sometimes, renders teeth difficult to extract, having the effect of rivetting them in their sockets—the ends of the fangs being increased double or triple their natural size. I extracted the first upper molar from the jaw of a delicate woman—two of the roots, after complete separation, united at their joints. I have extracted many, which seeming to have reached the anterior, turned horizontally, but only one in which a complete bony union had taken place without apparent exostosis.

Exostosis rarely if ever occurs on the roots of teeth while the crown is sound, but often in roots where the crown has decayed off, and although the pulp is destroyed, yet a portion of the nerve is alive, giving much pain. I extracted from the mouth of one patient two molares, and four bicuspids of the upper jaw, the fangs were increased three times their natural size by firm bone. She had suffered much nervous irritation. In these cases the periosteum secretes and deposits bony matter, as in necrosis of the tibia and other bones. This deposit would be advantageous on the incisores when we pivot artificial teeth, but on such teeth it is rarely seen, they are more frequently the subjects of alveolar abscess, the teeth bathed in pus waste away, become blackened, loose and drop out. This effect is frequently brought about by the injudicious force used by some dentists, in setting pivot teeth, and here I would say that pivot teeth should first be accurately fitted with soft pine, and then make a similar pivot of well seasoned hickory, in this way the jar occasioned by putting in and taking out the tooth can be avoided. The insertion of the hickory pivot, if well made, can be done with the thumb and fore-finger—let the hole be dry, a slight tap, having a piece of end-wood intervening, will set it home. When a tooth is set in this manner, inflammation rarely follows, unless it has previously existed in the root.

The extraction of the deciduous teeth rarely require the art of the dentist—in their regular course the fangs are absorbed and the crowns drop off the gums about the advent of the permanent teeth. I think the absorption of their fangs is hastened by the pressure of the permanent teeth. We always find the absorption greater next them. We occasionally meet with the fangs of the temporary teeth having passed through the alveolus and gum, and cutting the lip, this is caused by the rapid growth of the permanent teeth. In these cases I slit the gum in the direction of the fang and pull it out.

Occasionally, it is necessary to extract a deciduous tooth to make room for a permanent, this happens when the permanent teeth are large and come forward before there is a corresponding growth in the maxilla, here the deciduous central incisores are loose or lost, but the permanent teeth, from their greater size, come forward in an angular position, and then, unless the laterals are extracted, permanent derangement will take place. I have seen one case in a man of thirty-five years, where a central incisor was so turned, that the back part presented anteriorly. I could not get the history of the case, but concluded, from the irregularity of the teeth, that it was caused by the deciduous teeth not having been removed at proper periods. Afterwards it is generally necessary to extract the cuspidati to make room for the laterals, and here the necessity for extraction ceases; the deciduous molares having been replaced by bicuspids, and the jaw expanded so as to accommodate the permanent cuspidati. Should, however, this not be the case, the first permanent molar being sound, a bicuspid on one or both sides must be extracted, for the cuspidati must be preserved; no mouth can look well without them.

The first permanent molares, or six-year teeth, are often mistaken by parents for deciduous, and they are often lost before the attention of the dentist is called to them. In an article sent you on the subject of filling teeth, I insisted on the propriety of filling deciduous teeth with tin; many valuable teeth would thereby be saved from their coming under the inspection of a dentist in time; much infantile suffering would be avoided, and many an unquiet night be spared the parent, and it is, doubtless, of importance to preserve the crown of the deciduous tooth until the permanent is ready to take its place.

Hæmorrhage sometimes occurs after the extraction of the permanent teeth, and it is represented as difficult to arrest. I have used with uniform success a pledget of cotton saturated with the juice of lemon, or when that could not be had, I

dampen the cotton and roll it in the powdered tartaric acid which is nearly as good; pressure can be applied by cutting a piece of cork to fit between the teeth from whence the other was drawn, and then with a bandage closing the teeth to keep in place.

Mr. McL. had been salivated—a portion of the alveolus of the superior maxilla exfoliated, including the dens sapientiæ. I removed it, but little blood followed at the time; eight or ten hours after, about midnight, I was sent for; I took a lemon in my hand and went to his room; he must have bled a pint, it looked like a gallon; the bed and floor was apparently covered; and much blood and saliva in basins, &c. I saturated a pledget of cotton with the lemon-juice, applied it, and staid with him half an hour. The blood coagulated, and immediately ceased to flow; nor was there any return.

Mrs. McD. had her teeth loosened by salivary calculus, so as to be painful in any attempt to bite. I extracted them; followed by a profuse flow of blood. I used a lint-compress, covered by cork, the flow continued. I saturated cotton with tartaric acid, applied it, and on it placed the bit of cork. I had no farther trouble with it. The gums healed, and I replaced her own teeth on gold plate.

Miss D——. Extracted lower molar, followed by a flow of blood, but not much more than is usual. A few hours after I was sent for; the flow was profuse; used the tartaric acid; again sent for, it had not succeeded, and required several applications. I attributed the difficulty to a state of fever in the system existing at the time, and which required medication.

I was led to the use of the acid from its use in uterine hæmorrhage. It seems to have a specific power in coagulating the blood.

